# Global trends of targeted therapy for hepatocellular carcinoma: A bibliometric and visualized study from 2008 to 2022

**DOI:** 10.1097/MD.0000000000034932

**Published:** 2023-08-25

**Authors:** Xuan-Ang Yang, Rong Jin, Lei-Ming Zhang, Dong-Jian Ying

**Affiliations:** a Health Science Center, Ningbo University, Ningbo, Zhejiang Province, China; b The Affiliated Lihuili Hospital of Ningbo University, Ningbo, Zhejiang Province, China.

**Keywords:** bibliometrics, global trend, hepatocellular carcinoma, targeted therapy, visualized study, VOS viewer

## Abstract

**Background:**

Hepatocellular carcinoma (HCC) is an exceedingly prevalent malignancy with an exceptionally poor prognosis. Targeted therapy is an effective treatment option for patients with advanced HCC. However, there have been no bibliometric analyses of targeted therapies for HCC.

**Methods:**

This study aimed to assess the current status and future directions of targeted therapy for HCC to provide future scholars with clearer research contents and popular themes. Methods: Literature on targeted therapy for HCC from 2008 to 2022 was obtained from the Web of Science (WoS) and assessed using bibliometric methodology. Additionally, the VOS viewer was applied in the visualization study to conduct bibliographic coupling, co-authorship, co-citation, and co-occurrence analyses of publications.

**Results:**

A total of 10,779 papers were subsequently selected. Over the past 15 years, there has been a progressive increase in the number of publications on an annualized basis. China released the most publications in the field, whereas the United States had the highest H-index. Cancers published the most papers. Fudan University had the greatest sway in this area. Studies could be divided into 5 clusters: “Gene and expression research,” “Mechanism study,” “Nanoparticle study,” “Targeted drug research,” and “Clinical study.”

**Conclusions:**

In the upcoming years, more papers on targeted therapy for HCC are expected to be released, demonstrating the potential for this topic to flourish. Particularly, “Clinical study” is the following trendy topic in this field. Other research subfields may likewise exhibit a continuous tendency towards balanced development.

## 1. Introduction

Hepatocellular carcinoma (HCC) is the sixth most common malignancy and the fourth leading cause of mortality from cancer worldwide.^[1,2]^ Notably, it accounts for approximately 75% of all cases of primary liver cancer.^[1,3]^ Globally, the incidence of HCC is increasing annually, and the fatality rate is extremely high. In 2019, there were approximately 747,000 cases and 480,000 fatalities worldwide.^[4]^ Among the global health issues, it remains a situation that cannot be disregarded. Geographically, the incidence of HCC varies, although the majority of cases are found in less developed areas such as Eastern Asia (54.8%) and South-Eastern Asia (10.8%) identified.^[5]^ As a result of the enormous population base and rising HCC prevalence, China has the most patients with HCC.^[1]^ In China, chronic hepatitis B is the most potential hazard factor associated with the pathogenesis of HCC, with additional common causes including alcoholic liver disease, nonalcoholic fatty liver disease, metabolic syndrome, aflatoxin ingestion, and hereditary factors. In contrast, in certain Western nations, alcoholic cirrhosis, nonalcoholic fatty liver disease, and chronic viral hepatitis C are the primary drivers.^[6,7]^

Patients with HCC have a variety of therapeutic options, including surgical excision, liver transplantation, percutaneous ablation, radiotherapy, and transarterial and systemic therapy.^[8]^ The stage of HCC substantially influences the treatment strategy. To date, over 10 cancer staging systems, including the Barcelona Clinic Liver Cancer (BCLC) staging system, the Tumor-Node-Metastasis staging system, the Hong Kong Liver Cancer staging system, and the China liver cancer staging system, have been provided to evaluate the prognosis of HCC or to select the most appropriate course of therapy.^[9–11]^ In approximately 40% of patients with early HCC (BCLC-0 or BCLC-A), surgical treatment may induce a clinical cure. However, regrettably, the symptoms of HCC are rarely typical enough to attract timely attention. When patients with HCC receive a diagnosis, they are generally already at an advanced stage of the disease, lose the opportunity for radical surgery, and commonly failed to completely achieve their intended objectives of recovery. Targeted therapy is an effective alternative for such patients, prolonging their survival time and improving their prognosis.^[12]^ Targeted therapy is primarily indicated for patients afflicted with locally progressive or advanced HCC, who are unsuitable for surgery or transarterial chemoembolization. Furthermore, it is recommended for individuals in stages the China liver cancer IIIa and IIIb, experiencing vascular infiltration or extraneous hepatic tumor dissemination, as well as patients with tumor thrombosis in the main portal vein or inferior vena cava, and those confronted with tumor relapse or progression following surgery or transarterial chemoembolization. Based on the underlying mechanism, it can be categorized into antiangiogenic agents and multitarget tyrosine kinase inhibitors.^[13–15]^ The most commonly used first-line targeted medications for HCC are sorafenib and lenvatinib, along with others which include regorafenib, cabozantinib, ramucirumab and so forth.^[16,17]^ Sorafenib is a small tyrosine kinase inhibitor that decrease the activity of receptors for Raf kinase, vascular endothelial growth factor, and platelet-derived growth factor.^[18]^ Although it is the most commonly used targeted therapy for HCC, reviews have indicated that its administration only marginally enhances patient survival by several months.^[19,20]^ Hence, to attain superior therapeutic achievements, stronger medications as well as more thorough and sophisticated clinical trials are required. Moreover, Combination therapy may be superior to single-drug therapy in terms of benefits.^[21]^ A great deal of hope is given to patients with advanced HCC owing to the widespread implementation of targeted therapy in clinical practice. In light of this, it is indispensable to analyze the current state of targeted therapy for HCC on a global scale.

Publications are crucial criteria for measuring research trends and contributions which could reflect the significance of a particular subject. Bibliometric analysis is a statistical procedure used to analyze a large number of publications in terms of their qualitative and quantitative characteristics. It has been applied to scrutinize the scientific output productivity of researchers, journals, institutes, and countries in distinct academic subjects, to analyze research trends and long-term priorities within each discipline, and to determine policy decisions and clinical practice guidelines.^[22–24]^ Furthermore, the validity of this approach has also been demonstrated successfully with regard to assessing trends in spine health, heart failure, coronavirus disease 2019, diabetes, and breast cancer research. According to our knowledge, nonetheless, neither the volume nor the caliber of research into targeted treatments for HCC have been reported. Consequently, the primary objective of this research was to assess in-depth the current situations and future directions of targeted therapy research for HCC, to provide future scholars with clearer research contents and popular themes, so that they can reduce detours, enhance their scientific research capacity more efficiently, and ultimately improve the overall work efficiency in this domain, in order to contribute to the field vigorous progress.

## 2. Materials and methods

### 2.1. Data source

While there are several other databases available for literature search and bibliometric analysis, such as Scopus and Google Scholar,^[25]^ for this investigation, Web of Science (WoS) (SCI-Expanded) is our top choice because it is widely regarded as the most suitable database for bibliometrics.^[26]^

### 2.2. Search strategies

All publications were searched for in WoS from database inception to December 2, 2022. Publications were collected using the following search terms: theme = hepatocellular carcinoma AND theme = targeted therapy AND publishing year = (2008–2022) AND Language = (English) AND Document types = (ARTICLE OR REVIEW). To avoid daily updates, retrieval was carried out on the same day (December 2, 2022). It was not necessary to obtain informed consent because the non-personal informational data were all secondary. This methodological study was waived for ethical approval by the Ethics Committee of the Affiliated Lihuili Hospital of Ningbo University, as no patients were involved.

### 2.3. Data collection

To guarantee the reliability of the research, 2 authors independently selected the literature and extracted the data. Any issues that arose were resolved through discussion and negotiation. From the WoS database, the complete contents of each document, including title, year of publication, writer, nation, affiliation, journal, keywords, abstract, and many more, were obtained as TXT files and inputted into Microsoft Excel 2021. Finally, GraphPad Prism5 was used to further analyze the data.

### 2.4. Bibliometric analysis

Based on its inherent functionality, WoS was able to retrieve pivotal characteristics of qualifying articles. H-index: A professor or country has produced H papers, and other articles have cited their studies a minimum of H times. It can demonstrate the volume of literature and pertinent citations, and assess the worth and popularity of academic project.^[27,28]^ Additionally, the relative research interest was derived by dividing the number of papers on a specific topic each year by the total number of documents published throughout all domains during an identical timespan.^[29]^ By adopting a logistic regression equation, the time curve to incorporate the time trend of papers was drawn using R programming language. In this equation, year is symbolized by the independent variable x, and the overall number of publications is symbolized by the dependent variable Y.

### 2.5. Visualized analysis

The VOS viewer is currently the most comprehensive and suitable tool for serving the purpose of bibliometric visualization and journal analysis.^[30]^ In this study, bibliographic coupling, co-authorship, co-citation, and co-occurrence analyses were performed using the VOS viewer. The dimensions of each node and the thickness of the lines connecting them reveal the frequency of their constituent parts and the potency of the connections between various nodes. The shapes of the nodes correspond to the individual sizes of the nodes, whereas the nodes themselves represent various elements.^[31,32]^

## 3. Results

### 3.1. Global trends in publishing

The future prospects of research in this field are expected to witness a continual augmentation, facilitating the accumulation and dissemination of knowledge, fostering international collaboration and exchange of experiences, leading to the exploration and validation of innovative therapeutic approaches, offering a wider array of treatment options, enhancing the survival rates of patients, and providing theoretical guidance for clinical practices in HCC.

#### 3.1.1. Worldwide publications count.

**Figure 1.**
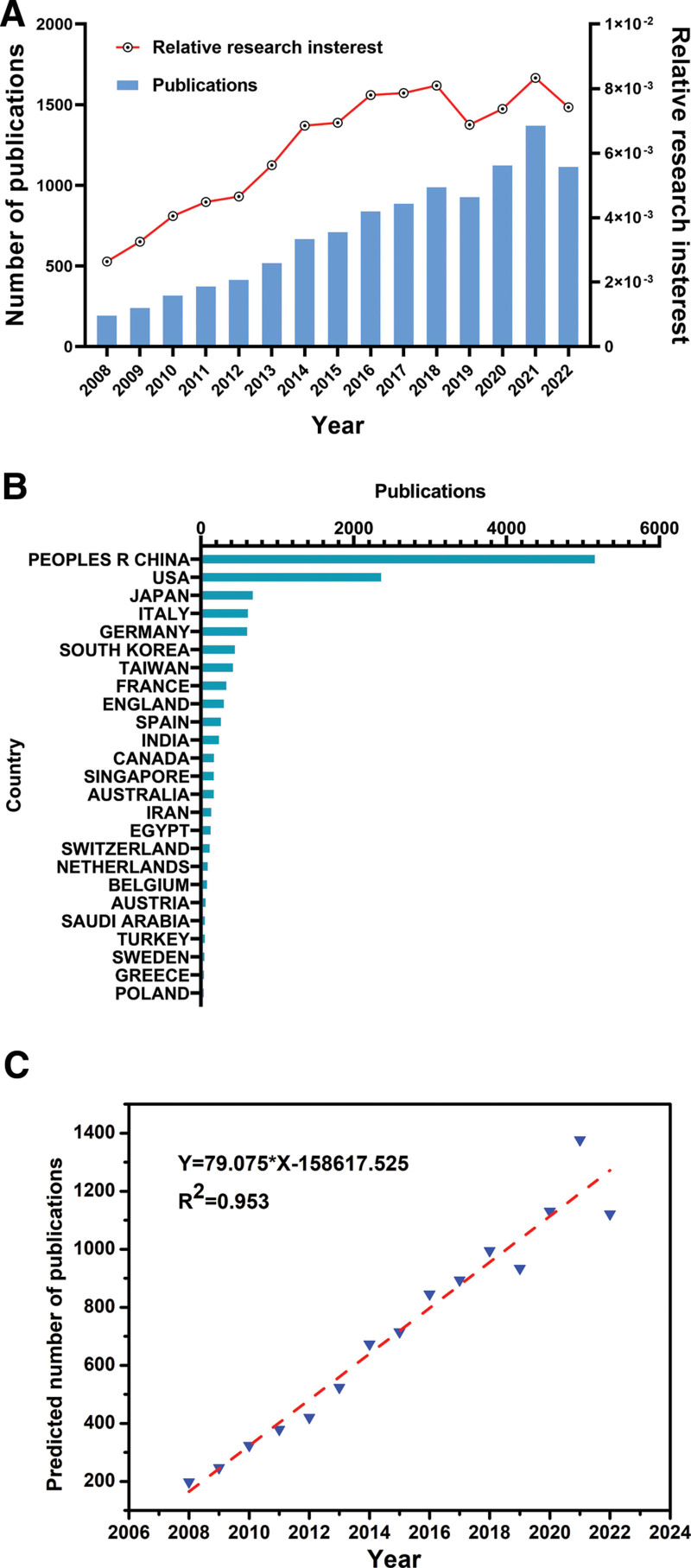
The global state of research on targeted therapy for HCC and the nations contributing. (A) The entire number of articles on targeted therapy for HCC published worldwide. The blue bars represent the amounts of single-year publications. (B) The sum of targeted therapy for HCC-related papers from the leading 25 nations. (C) The model-fitting curve for the incremental tendency in publications on a global scale to forecast the amount of literature towards the future. HCC = hepatocellular carcinoma.

After submitting the search parameters, 10,779 publications between 2008 and 2022 were gathered. Over the past few years, there has been a progressively expanding number of publications on an annualized basis. In addition, considering the number of articles by year, most publications (1377, 12.78%) were published in 2021 (Fig. 1A).

#### 3.1.2. National and regional contributions.

In the realm of targeted therapy for HCC, pertinent literature has been published in 107 nations and territories. As for the quantity of papers released (Fig. 1B), China contributed the most (5179, 48.05%), followed by the USA (2381, 22.09%), Japan (703,6.52%), Italy (638, 5.92%), and Germany (629, 5.84%).

#### 3.1.3. Trends in global publications.

To estimate the prospective change trend of the worldwide number of publications on this subject, the logistic regression equation was applied to demonstrate the temporal curve of the publication count across all years. The incremental tendency in publications on a global scale is generally consistent with the model-fitting curve Y = 79.075x − 158617.525 (*R*²= 0.953), as illustrated in Figure 1C, indicating that the annual article volume in this sector is expected to proliferate linearly.

### 3.2. Publishing standards among various nations

The research achievements of China and the USA in this domain bear substantial academic influence, exerting a positive impact on the clinical treatment of HCC in the future. They have propelled the development of novel targets and delivery approaches, thereby offering more efficacious and less adverse treatment regimens. Furthermore, they have stimulated investigations into the pharmacokinetics and excretion of drugs for targeted therapy of HCC, leading to the optimization of drug utilization and dosage adjustments.

#### 3.2.1. Total citation frequency.

**Figure 2.**
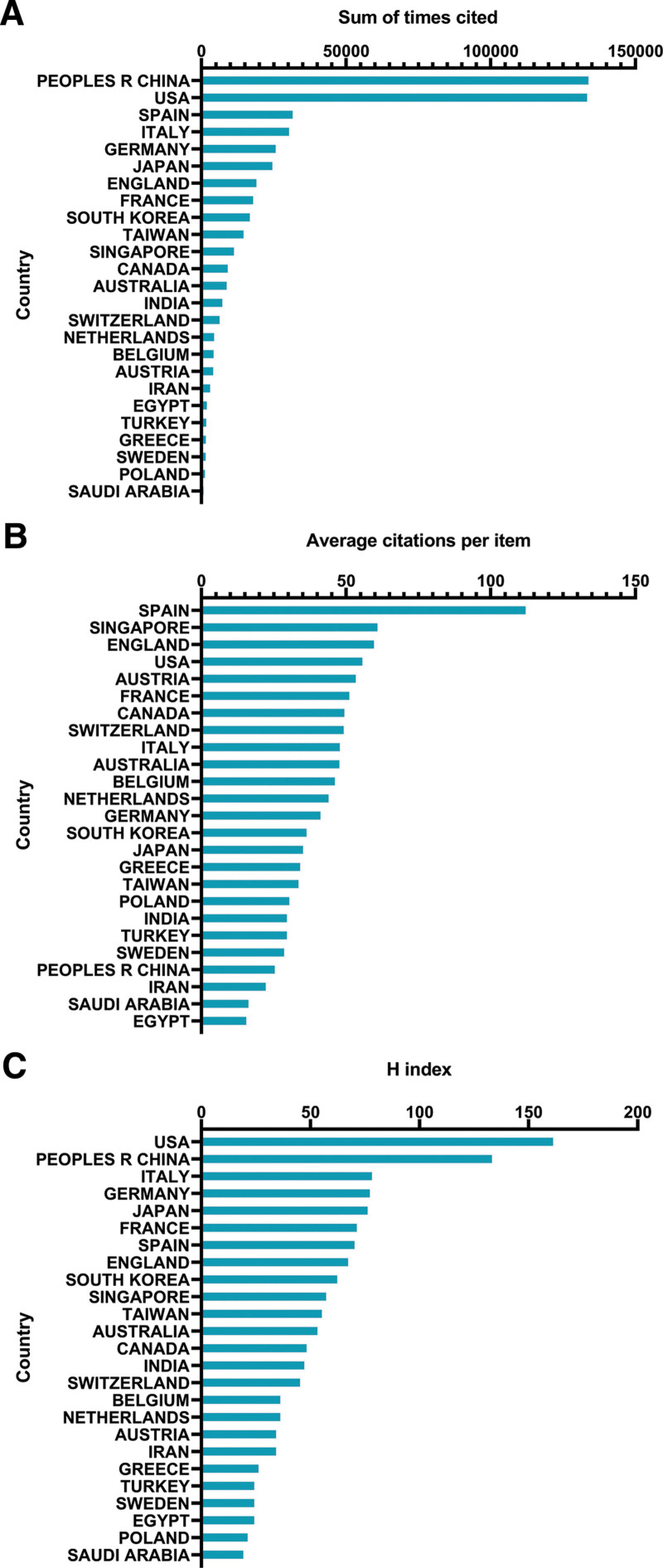
H-index scores and citation frequency for various nations. (A) The leading 25 nations of total citations of targeted therapy for HCC. (B) The average volume of citations per publication for writings from distinct nations. (C) The H-index of articles in various countries. HCC = hepatocellular carcinoma.

Among all the articles involved, China received 134,358 total citations, followed by the USA came in second with 133,908 total citations, Spain (32,101), Italy (30,904), and Germany (26,276) (Fig. 2A).

#### 3.2.2. Average citation frequency.

The highest average citation frequency occurred in publications from Spain (112.64), followed by Singapore (61.45), England (60.26), the USA (56.24), and Austria (53.97) (Fig. 2B).

#### 3.2.3. H-Index.

The relative writings of the USA had the greatest H-index (162), followed by China (134), Italy (79), Germany (78), and Japan (77) (Fig. 2C).

### 3.3. Analysis of global publication

Specific journals, focused research directions, prolific authors, and institutions have a positive influence on the future clinical treatment of HCC. They aid physicians in gaining a deeper understanding of patients’ varied responses to targeted medications, facilitating the adjustment of treatment strategies and guiding the development of personalized therapeutic approaches.

#### 3.3.1. Journals.

**Figure 3.**
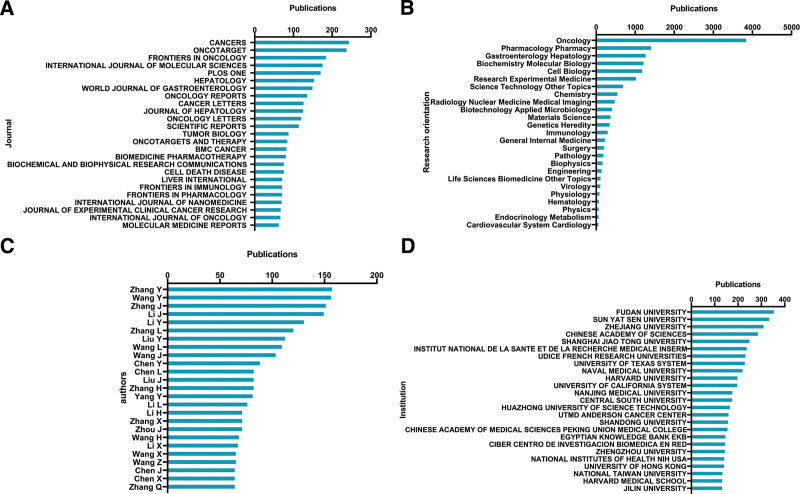
Scholarly journals, research orientations, writers and outstanding institutions about targeted therapy for HCC. (A) The top 25 journals for articles on targeted therapy for HCC. (B) The top 25 research orientations, together with the quantity of papers published in each. (C) The top 25 most prolific writers. (D) The top 25 most prominent institutions, as well as the amounts of articles produced by each. HCC = hepatocellular carcinoma.

*Cancers* published the most studies, with 245 publications outnumbering other journals. The second-placed *Oncotarget* had 239 papers. Furthermore, 186 articles on targeted therapy for HCC were documented in *Frontiers in Oncology*, 177 in the *International Journal of Molecular Sciences*, and 172 in *Plos One*. Figure 3A presents an overview of the top 25 journals based on their number of scholarly papers.

#### 3.3.2. Research orientations.

Figure 3B depicts the top 25 research directions pertinent to targeted therapies for HCC. The most favored main research interests were oncology (3855 papers), pharmacology pharmacy (1425 papers), gastroenterology hepatology (1282 papers), biochemistry molecular biology (1232 papers), and cell biology (1200 papers).

#### 3.3.3. Authors.

The top 25 authors contributed 2373 articles and reviews, accounting for 22.02% of the literature in this discipline. With 158 articles, Zhang Y was ahead of the competition, followed by Wang Y and Zhang J, who had 157 and 152 writings, respectively (Fig. 3C).

#### 3.3.4. Institution output.

Figure 3D exhibites the list of the top 25 contributing institutions. With 356 papers, Fudan University issued the most, Sun Yat-sen University emerged second (337 papers), and Zhejiang University ranked third (312 papers).

### 3.4. Bibliographic coupling analysis

The similarity between articles was measured via bibliographic coupling, and a comparable theme appeared to be shared by these publications. The strength of the links between journals, institutions, and nations in each study was investigated using VOS viewer. Journals, institutions, and countries with robust total link strength possess the capacity to offer crucial research resources and collaborative partnerships to researchers and clinical practitioners. Such associations aid them in accessing the latest scientific achievements and treatment advancements, subsequently integrating them into practical applications, thereby enhancing the level of clinical care.

#### 3.4.1. Journals.

**Figure 4.**
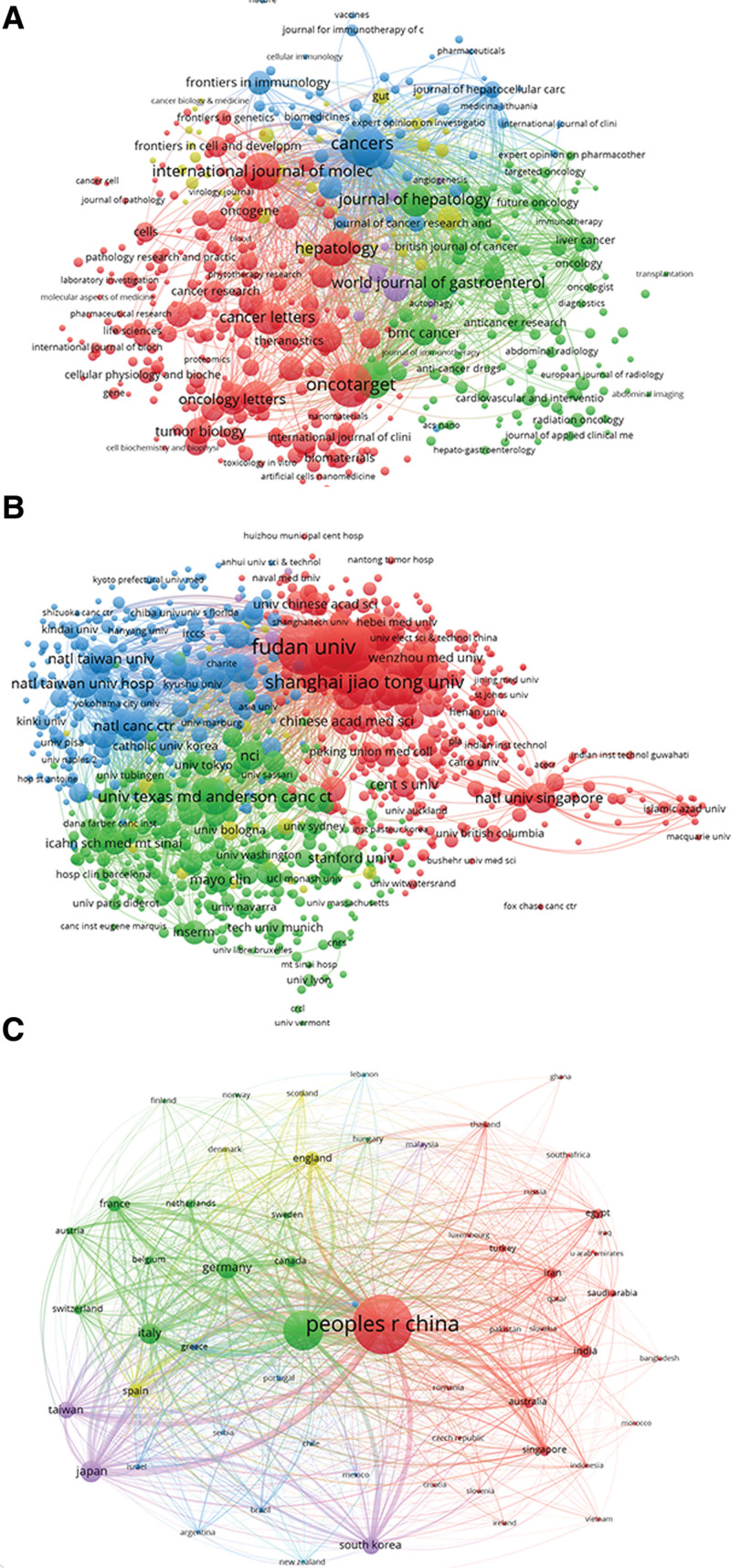
Analysis of worldwide studies on targeted therapy for HCC using bibliographic coupling. (A) The entire list of 453 relevant journals on targeted therapy for HCC. (B) The entire list of 977 institutions on targeted therapy for HCC. (C) The entire list of 60 countries on targeted therapy for HCC. The line connecting 2 nodes indicated how resemble the corresponding journals, institutions, and nations were to one another. Journals/institutions/nations with thicker lines were closer linked. HCC = hepatocellular carcinoma.

Taking the total link strength into account, as depicted in Figures 4A, 453 identified journals were detected (each journal requires no fewer than 5 papers). Five journals with large total link strength were as follows: *Cancers* (448,303 times), *World Journal of Gastroenterol* (258,335 times), *Frontiers in Oncology* (243,215 times), *Oncotarget* (221,201 times), and *Journal of Hepatology* (200,675 times).

#### 3.4.2. Institutions.

For the total link strength, 977 institutions were retrieved, each of which possessed 5 or more articles in this domain. Below is a list of the 5 institutions that held the greatest total link strength: Fudan University (852,674 times), Zhejiang University (699,895 times), Sun Yat-sen University (695,784 times), National Taiwan University Hospital (528,796 times), and University of Hong Kong (488,788 times) (Fig. 4B).

#### 3.4.3. Countries.

The VOS viewer examined publications from 60 distinct nations (a number of publications from a nation that had been utilized at least 5 times) (Fig. 4C). The top ranking was China (4,571,240 times), followed by the USA (3,970,463 times), Italy (1,702,605 times), Japan (1,603,490 times), and Germany (1,259,971 times).

### 3.5. Co-authorship research

A co-authorship study demonstrated how various items were interrelated relying on the number of publications they had in common. Using the VOS viewer, the total link strengths of authors, institutions, and countries were identified. These findings hold significant instructive value for the future clinical treatment of HCC, as they can foster collaborative research, optimize resource allocation, facilitate cross-border cooperation, and promote knowledge exchange. Moreover, they serve as guiding beacons for future research directions and focal points, providing enhanced support and advancement for the treatment of HCC.

#### 3.5.1. Authors.

**Figure 5.**
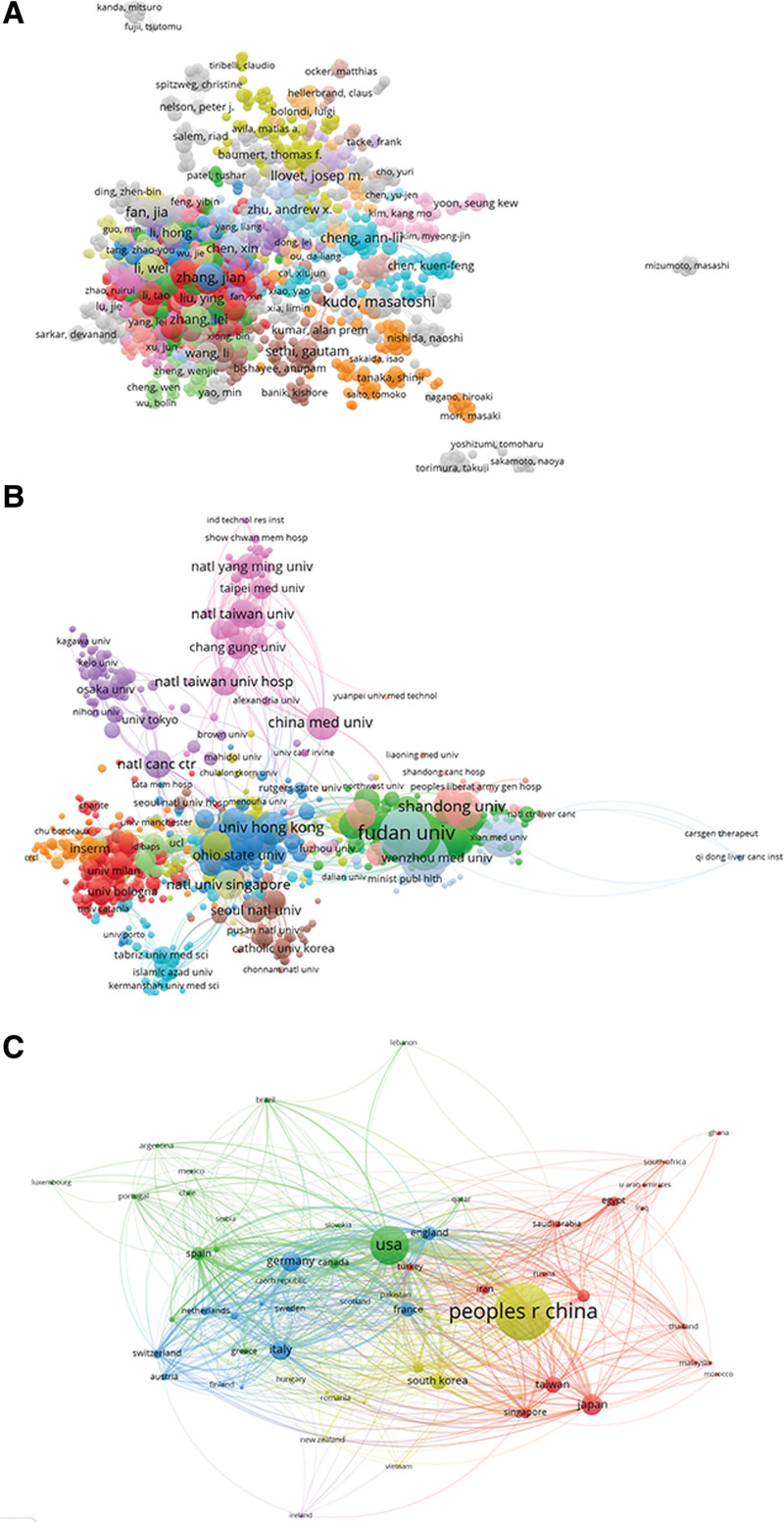
Analysis of worldwide studies on targeted therapy for HCC using Co-authorship. (A) The entire list of 935 writers co-authorship analysis on targeted therapy for HCC. (B) The entire list of 975 institutions co-authorship analysis on targeted therapy for HCC. (C) The entire list of 60 countries co-authorship analysis on targeted therapy for HCC. The co-authorship frequency is depicted by the size of the spots. The collaboration between 2 writers, institutions, or nations was portrayed by the line connecting the 2 spots in the image. Thicker lines indicate tighter collaboration between 2 authors/institutions/countries. HCC = hepatocellular carcinoma.

There were 935 writers with at least 5 publications on the topic that were revealed with the total link strength, as exemplified in Figure 5A. According to the total link strength, the following 5 writers were at the top: Fan J (253 times), Zhou J (239 times), Kudo M (178 times), Llovet JM (152 times), and Sethi G (128 times).

#### 3.5.2. Institutions.

The final inclusion of 975 institutions with more than 5 publications as a minimum restriction is shown in Figure 5B. The Chinese Academy of Sciences (506 times) was ranked first, followed by Fudan University (447 times), Sun Yat-sen University (411 times), Shanghai Jiao Tong University (360 times), and the National University of Singapore (347 times).

#### 3.5.3. Countries.

With the VOS viewer, distinguished publications (defined as the bare minimum quantity of a country articles that were cited more than 5 times) among the 60 nations were evaluated. The top 5 nations in terms of total link strength were the USA (2042 times), China (1129 times), Germany (769 times), Italy (657 times), and England (563 times) (Fig. 5C).

### 3.6. Co-citation research

In accordance with a study on co-citation, the relationship between 2 items can be established by counting the number of times they are quoted in the same document. VOS viewer was used to assess the total co-citation link strength of articles and journals. Highly cited articles and journals represent research achievements and academic perspectives of paramount significance. Clinical practitioners and researchers can draw inspiration from these research findings, thereby enhancing the quality and impact of their clinical practice and research endeavors. They aid in uncovering pivotal research topics, hot-button issues, and academic trends within the field, providing guidance and direction for future clinical treatments.

#### 3.6.1. Publications.

**Figure 6.**
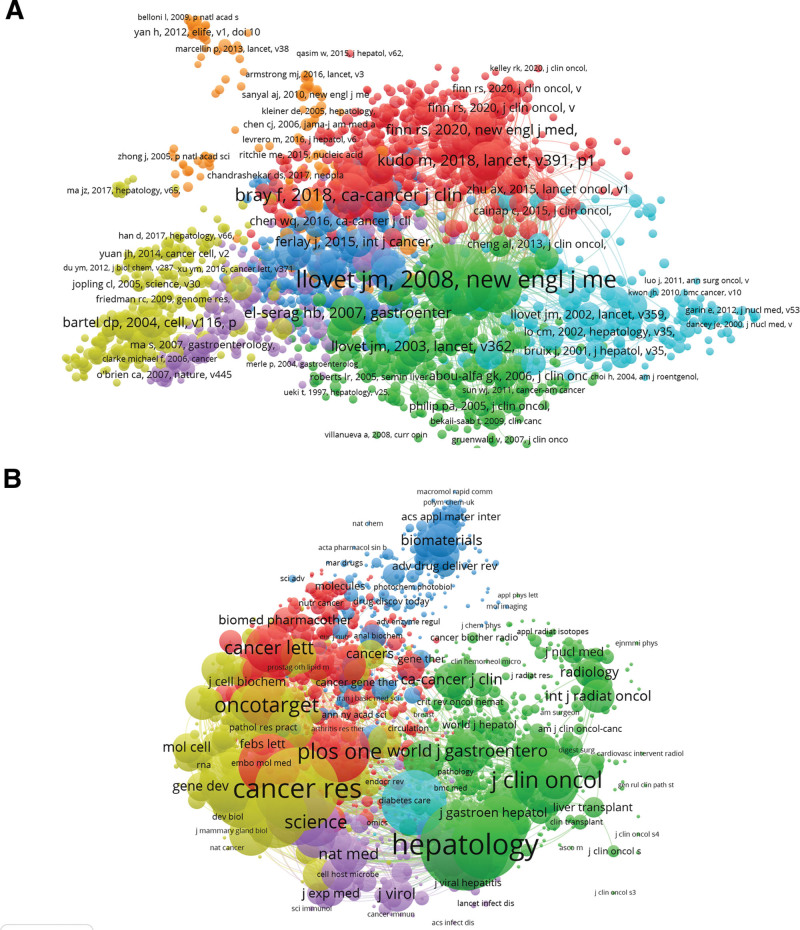
Analysis of worldwide studies on targeted therapy for HCC using Co-citation. (A) The entire list of 2014 included publications by co-citation analysis on targeted therapy for HCC. (B) The entire list of 1989 involved journals by co-citation analysis in the domain. The citation frequency is signified by the point sizes. Diverse points were connected by a line to denote that they were referenced in 1 paper. The link between 2 articles is stronger when a line is shorter. The same color of the points designates the same study domain to which they belong. HCC = hepatocellular carcinoma.

The analysis comprised 2014 papers (defined as references cited with a minimum of 20 citations or more), which were processed via the VOS viewer (Fig. 6A). The leading 5 papers with large total link strength were mentioned below: Llovet JM, 2008, N Engl J Med^[33]^ (31,360 times); Cheng AL, 2009, Lancet Oncol^[34]^ (18,699 times); Bruix J, 2017, Lancet^[35]^ (11,814 times); Kudo M, 2018, Lancet^[36]^ (11,786 times); El-Khoueiry AB, 2017, Lancet^[37]^ (11,614 times).

#### 3.6.2. Journals.

The journal was deemed to have at least 20 citations from a single source. Figure 6B reflected that a total of 1989 journals satisfied this requirement. The leading 5 journals with the mightiest total link strength were mentioned below: *Hepatology* (2,182,505 times), *Cancer Research* (2,178,735 times), *Journal of Hepatology* (1,352,393 times), *Nature* (1,326,079 times), and *PloS One* (1,290,580 times). These were just a few of the periodicals that have received numerous citations.

### 3.7. Co-occurrence research

**Figure 7.**
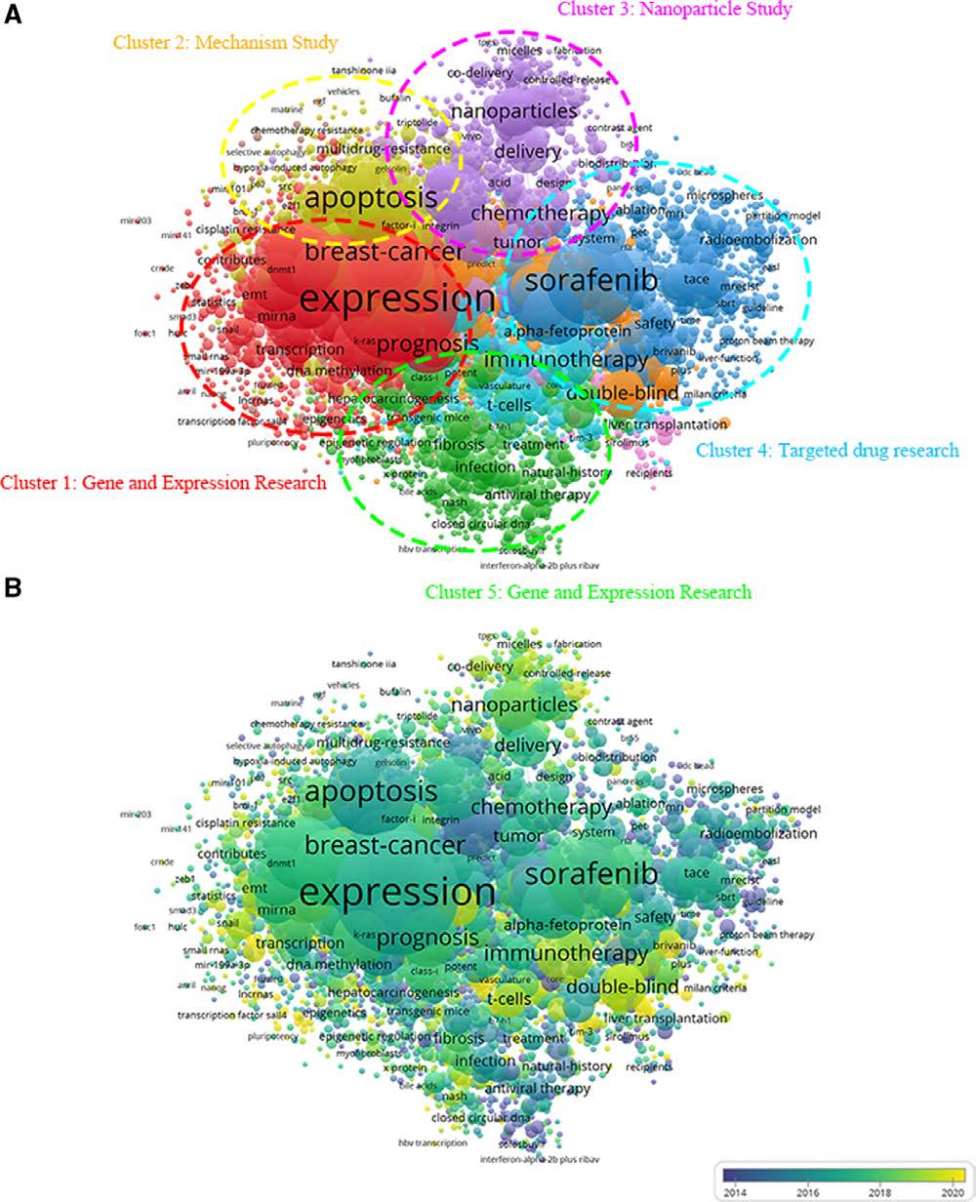
Co-occurrence analysis on targeted therapy for HCC. (A) The entire list of keywords in this study about targeted therapy for HCC; The frequency was denoted based on the size of the points, and the keywords were separated into 5 clusters: Gene and expression research (color red), Mechanism study (color yellow), Nanoparticle study (color purple), Targeted drug research (color blue) and Clinical study (color green). (B) Keywords were distributed in accordance with how frequently they appear. The color blue suggests an earlier appearance of the keywords, whereas the color yellow indicates a later occurrence. HCC = hepatocellular carcinoma.

A co-occurrence study proved the association between items by relying on how frequently they appeared together in publications. Its purpose is to reveal prominent study topics and directions, and it also serves a crucial function in monitoring how scientific research is progressing.^[38,39]^ Through the VOS viewer, keywords, defined as terms used more than 5 times across all publications, were reviewed. The 3260 obtained keywords were roughly grouped into 5 clusters, as seen in Figure 7A, including “Gene and expression research,” “Mechanism study,” “Nanoparticle study,” “Targeted drug research” and “Clinical study.” The most commonly used keywords in the “Gene and expression research” cluster were expression, transcription, prognosis, deoxyribonucleic acid methylation and micro ribonucleic acid. In the “Mechanism study” cluster, the leading terms were: apoptosis, factor-i integrin, multidrug-resistance, gelsolin and hypoxia-induced autophagy. In the “Nanoparticle study” cluster, the strong topics were: nanoparticles, tumor, chemotherapy, delivery and controlled-release. In the “Targeted drug research” cluster, the most generally mentioned keywords were sorafenib, safety, time, the modified Response Evaluation Criteria in Solid Tumors, and brivanib. In the “Clinical study” cluster, the principal keywords were: treatment, infection, double-blind, fibrosis and antiviral therapy. These conclusions demonstrate the distribution of research directions in publications on targeted therapies for HCC.

The VOS viewer encoded keywords with various hues in Figure 7B based on the average time when they appeared throughout all articles that had been published. In contrast to the yellow-colored keywords, which appeared later, blue-colored keywords emerged earlier. As a consequence of the findings, prior to 2014, the majority of studies in the field early stages were on “Nanoparticle study” and “Targeted drug research.” However, current trends suggested that “Gene and expression research,” “Mechanism study” and “Clinical study” were garnering more attention as this field of study developed and matured. There were several variations in research hotspots. Additionally, this implies that future research on targeted therapy for HCC would follow a variety of developmental trends.

Co-occurrence analysis can assist researchers in identifying the primary research directions and focal points within the field, enabling them to comprehend the forefront areas of current investigations and predict future developmental trends. These insights are instrumental in guiding the development and refinement of clinical treatment methods for HCC based on research progress. Notably, the domains of “Gene and expression research,” “Mechanism study,” and “Clinical study” have gained popularity. For instance, by examining pathogenic genes and aberrant activation of signaling pathways, targeted therapeutic drugs can be developed. The efficacy of these treatments can be determined through extensive clinical trials, leading to the creation of personalized treatment plans for patients.

## 4. Discussion

### 4.1. Trends in targeted therapy for HCC

Bibliometric and visualized studies can depict the current state of research in associated professional disciplines and forecast rising hotspots and tendencies.^[38]^ Therefore, our objective was to assess targeted therapy for HCC research using bibliometric and visual methods, and to further demonstrate its future trajectories from the perspectives of papers, authors, contributing countries, institutions, and research priorities. With the data from our study, we realize that since 2008, there has been an annual growth in the number of publications in this sector that is statistically significant, indicating a surge in research interest in the field. In total, 107 countries have produced pertinent literature in this domain. Based on the available data, it is anticipated that in the future, there will be greater interest in targeted therapy for HCC and more publications on the topic. The existing favorable outcomes will in turn allow researchers to conduct more high-quality studies.

### 4.2. Status and quality of global publications

The overall citation counts, average references for each article, and a nation H-index are indicators of the standard of its publications and their influence on academia.^[40]^ In our studies, from the standpoint of the total number of papers released and the entire citation frequency, China contributed the most to worldwide scholarships. Within this sector, the USA possessed the strongest H-index, as well as high total and average citation frequencies. Therefore, we considered China and the USA to be the top 2 nations in the area of targeted therapy for HCC. However, the average citation frequency of China only ranked 22nd, which might be a result of the disparity between the quality and volume of the literature there. In China, the system for evaluating academic work prioritizes quantity over quality.^[41]^ Because of this system, Chinese academics have paid too much attention to the quantity and pace of publications rather than the quality of their work. Nevertheless, in recent years, with increasing investment in scientific research finances and energy, it is feasible to expect rapid development in the caliber of Chinese scholars’ research, closely keeping up with the rate of certain developed countries. Spain had the highest average citation frequency, which plays an essential role in this subject.

In terms of journals, *Cancers, Oncotarget, Frontiers in Oncology, International Journal of Molecular Sciences*, and *Plos One* published the most articles on targeted therapy for HCC. The principal outlets for publishing future studies in this discipline are the journals mentioned in Figure 3A.

The majority of the most prominent 25 institutions belonged to the leading 5 countries with the most publications, implying that enhancing a nation academic standards hinges on the establishment of world-class scientific institutions, and that national attention could promote the foundation and strengthening of research institutions. Among the most prolific authors in this field, Zhang Y, Wang Y, and Zhang J topped the list. The leading 25 scholars who have published the most articles can be regarded as pioneers of studies on this topic, and they are more likely to have a significant impact on the forthcoming advancement of this field.

Through bibliographic coupling analysis, the current study illustrated a comparable association among articles based on journals, institutions, and countries. Bibliographic coupling, which occurs when various studies incorporate identical references in their published works, allows for a greater understanding of how authors create connections between relevant materials. Our findings showed that *Cancers* was the most relevant journal in this area, and China held the top spot. The degree of cooperation among diverse writers, countries, and institutions was estimated through co-authorship research. The stronger the total link strength, the more probable writers, institutions, and nations are to collaborate. The outcomes of the co-authorship research in this study were Fan J, the Chinese Academy of Sciences, and the USA, respectively. By tracking how frequently different studies were referenced together, co-citation analysis was used to measure their influence. In light of the latest findings, the most commonly cited journal in this subject was *Hepatology*, which might be deemed as the source of seminal articles on HCC targeted therapy.

### 4.3. Research focus on targeted therapy for HCC

A co-occurrence analysis was conducted to evaluate and identify the relationships between items based on the number of co-occurrence papers. In addition, it is proposed as a promising approach for forecasting forthcoming trends and hot spots in linked scientific domains.^[42]^ As part of this study, we created a visual map of the co-occurrence relationships between the vital terms of all included papers. This was based on the co-occurrence map (Fig. 7A). “Gene and expression research,” “Mechanism study,” “Nanoparticle study,” “Targeted drug research” and “Clinical study” were the 5 potential research areas we have discovered. (Even if such an outcome is consistent with the professional general sense, this analysis could help clarify the path of subsequent study). This chart gives us a clearer idea of the future tendencies in this area that are most plausible. On the central area of the co-occurrence map, terms such as sorafenib, expression, apoptosis, prognosis, and double-blind were indicated with larger icons. It is imperative to continue to expand investment and standards for high-quality research on targeted therapy for HCC, as these 5 directions correspond to the research hotspot.

Items were marked on the overlay visualization map with various hues depending on the average time at which the keywords occurred.^[43]^ With the exception of color, this is analogous to the co-occurrence map. However, it has a superior ability to track the advancement of academic trajectories and anticipate upcoming themes. Diverse colors in the overlay visualization map of this study (Fig. 7B) reflect different publication years. The findings proved that yellow and light green patterns made up a significant portion of the “Clinical study,” indicating that after 2018, academics would concentrate more attention to this portion of the research content and investment, which would be potential to become the hot and focus of research in the coming years. “Clinical study” of targeted therapy for HCC have been widely undertaken, notably in freshly developed targeted medication clinical trials and combination therapy.^[44–47]^ Overall, these 5 components, which ranged in color from blue to yellow, showed that there had been a trend toward balanced and orderly development in each cluster research during previous years. Furthermore, the hot spots within each cluster are also undergoing diversified transformations, and could experience substantial development in the future.

In accordance with the results of this analysis, targeted therapy research for HCC has experienced a sharp increase in publications every year since 2008. This encouraging trend can motivate additional studies. Bibliometrics and visualized analysis can inform scholars about prominent nations, notable writers, and renowned institutions in the subject, as well as their collaborations and academic significance. With this expertise, novice researchers can easily pick up cutting-edge knowledge and worthwhile discoveries that are specific to their own requirements. They can also provide an intuitive visual picture and a more comprehensive understanding of the topic. In addition, the identification of trends and future study orientations using co-occurrence analysis and overlay visualizations helps researchers select topics and aids funding institutions in creating profit investment plans for academics to provide financial assistance and support for their work.

### 4.4. Molecular targeted agents for HCC

As of the present moment, the drugs approved by the U.S. Food and Drug Administration for targeted therapy in HCC encompass sorafenib, lenvatinib, regorafenib, cabozantinib, and ramucirumab.^[48]^

Sorafenib, the earliest approved targeted therapy for HCC, is a tyrosine kinase inhibitor that can extend the mean overall survival (OS) of patients with advanced HCC by 2.8 months.^[33]^ However, it presents certain challenges, such as adverse reactions like hypertension, diarrhea, and skin rash, and the development of drug resistance in patients prolonged use gradually diminishes its efficacy. Lenvatinib, as an another first-line drug in the realm of targeted therapy for HCC, exhibited a median OS 16.8 months in a clinical trial involving 940 participants, surpassing Sorafenib 15.3 months.^[49]^ Moreover, multiple investigations have attested to the prolongation of OS in patients treated with lenvatinib compared to those treated with sorafenib.^[36,50]^ Nonetheless, lenvatinib grapples with challenges such as drug resistance, as well as the occurrence of complications like hand-foot syndrome and decreased appetite. Additionally, the issue of determining the most suitable patient selection for optimal response remains a complex puzzle. Regorafenib, an agent of the second-line HCC targeted therapy, finds its suitability in patients who have previously received sorafenib treatment but experienced disease progression. In a clinical trial, in comparison to the placebo group, individuals who received regorafenib witnessed an increase of 2.8 months in their median OS.^[35]^ Its adverse reactions bear resemblance to those of sorafenib. While these drugs demonstrate therapeutic efficacy in HCC treatment, each faces unique challenges during therapy. These obstacles underscore the importance of personalized treatment, ensuring optimal treatment outcomes.

Furthermore, in recent years, the field of “Clinical Research in HCC Targeted Therapy” has garnered considerable attention. For instance, apatinib, a potent tyrosine kinase inhibitor, exerts its effects by targeting vascular endothelial growth factor receptor 2 to hinder angiogenesis. In a phase III clinical trial, apatinib demonstrated a substantial enhancement in the OS of pretreated advanced HCC patients compared to the placebo group.^[51]^ In addition, a phase II study revealed that the implementation of neoadjuvant dovitinib treatment prior to locoregional therapy presents a viable and feasible strategy.^[52]^ Besides, combination therapy bears superior advantages, striving to strengthen anti-tumor responses and elevate therapeutic efficacy through diverse mechanisms of action. In a phase Ib/II study, the combination of BLU-554 and CS1001 exhibited a secure and potent treatment for locally advanced or metastatic HCC.^[53]^ Additionally, in a phase II clinical trial, the effectiveness and safety of the combination of camrelizumab and apatinib during the perioperative period of resectable HCC are praiseworthy.^[54]^ These findings underscore the immense interest evoked by the combination of immunotherapy and targeted therapy as a joint therapeutic approach. We ardently believe that these novel treatment strategies can be subjected to more profound clinical trials, and expeditiously approved by the Food and Drug Administration for clinical application.

### 4.5. Future and application of targeted therapy for HCC

The aim of this study is to comprehensively assess the current status and future directions of targeted therapy research for HCC, in order to provide guidance for the clinical application of targeted drugs in the future. According to the findings of this study, we assert that it is imperative to enhance the international collaborative network in order to foster exchange and cooperation among diverse nations, institutions, and researchers. Such endeavors shall facilitate the exchange of experiences and knowledge, thereby collectively propelling the advancement of targeted therapy for HCC. Furthermore, with the burgeoning research interest in HCC targeted therapy, an increasing number of investigators will dedicate themselves to this field of study, consequently garnering support from more funding agencies. Consequently, they will have greater opportunities to undertake larger-scale clinical trials, employ rigorous research designs, conduct meticulous data analysis, and explore the mechanisms and development of novel therapeutic agents. By promoting collaboration between the scientific and medical communities, the translation from laboratory to clinical practice can be expedited, thus augmenting the reliability of treatment efficacy evaluation and accelerating the clinical application of targeted therapy for HCC. Moreover, through continuous exploration and analysis of the genomic information and tumor characteristics of HCC patients, the realization of precise personalized targeted therapy becomes feasible. Additionally, an in-depth comprehension of the molecular mechanisms of HCC may engender the development of more efficacious targeted drugs that selectively address specific tumor mutations and signaling pathways. These drugs can act more precisely on tumor cells, enhancing therapeutic efficacy while mitigating adverse effects on normal cells. Future clinical treatment for HCC may underscore the integration of multimodal therapies, necessitating the amalgamation of distinct treatment modalities such as surgery, radiotherapy, chemotherapy, targeted therapy, and immunotherapy, with the goal of eradicating tumors to the maximum extent and preventing recurrence.

As a result, we are confident that targeted pharmacological therapy will prosper and continue to be crucial for the management of HCC, offering patients with advanced HCC effective care and the potential to lengthen their lives.^[55]^

## 5. Strength and limitation

To the best of our knowledge, few studies have used bibliometric analysis of targeted therapy for HCC prior to this analysis. Although our investigation evaluated the present situation and prospects of targeted therapy for HCC through bibliometrics and visualization analysis and revealed research focus and cooperative relationships across various nations, writers and institutions, there still remain inescapable limitations. First, even if the included papers were sufficient to describe the current state of research, our data were gathered solely from the WoS database. Consequently, because of database bias, the selected literature could not be sufficiently extensive. In addition, due to linguistic bias, papers written in other languages might have been neglected since each publication indicated for inclusion was in English. Moreover, the analysis conclusions of this study may diverge from actual research findings in the real world, which would be another discrepancy. For instance, many freshly issued high-quality publications can fail to have adequate emphasis, owing to the short-term low citation frequency. Thus, in our daily academic research, we must continue to keep an eye on the current predominant analyses as well as additional non-English papers to retrieve the valuable information we need from multiple databases.

## 6. Conclusions

This research described the present academic quality and worldwide development trend of targeted therapy for HCC using bibliometric analysis. In this branch of research, both China and the USA have notably contributed, and are at the forefront of global research. Most articles on this theme have been published in the journal *Cancers*. In the coming years, more studies on targeted therapy for HCC are expected to be published, demonstrating the potential for this topic to flourish. Particularly, the “Clinical study” is the following trendy topic in this field. Other research subfields may likewise exhibit a continuous tendency towards balanced development. In summary, research on targeted therapy for HCC is an ongoing research hotspot, with targeted drugs continuously advancing and being synergistically combined with other treatments, thus enabling a more personalized and precise application in clinical practice. Such endeavors are beneficial to the health and prognosis of patients with advanced HCC. This still necessitates persistent efforts, and we believe that we shoulder heavy responsibilities.

## Author contributions

**Conceptualization:** Rong Jin, Dong-Jian Ying.

**Data curation:** Xuan-Ang Yang, Rong Jin, Lei-Ming Zhang.

**Funding acquisition:** Dong-Jian Ying.

**Investigation:** Xuan-Ang Yang, Lei-Ming Zhang.

**Methodology:** Xuan-Ang Yang, Rong Jin, Lei-Ming Zhang.

**Resources:** Dong-Jian Ying.

**Software:** Xuan-Ang Yang, Rong Jin.

**Validation:** Lei-Ming Zhang, Dong-Jian Ying.

**Visualization:** Xuan-Ang Yang.

**Writing – original draft:** Xuan-Ang Yang.

**Writing – review & editing:** Xuan-Ang Yang, Dong-Jian Ying.
